# Asparagine availability controls germinal centre B cell homeostasis

**DOI:** 10.1126/sciimmunol.adl4613

**Published:** 2024-12-13

**Authors:** Yavuz F. Yazicioglu, Eros Marin, Hana F. Andrew, Karolina Bentkowska, Julia C. Johnstone, Robert Mitchell, Zhi Yi Wong, Kristina Zec, Joannah Fergusson, Mariana Borsa, Iwan G. A. Raza, Moustafa Attar, Mohammad Ali, Barbara Kronsteiner, Izadora L. Furlani, James I. MacRae, Michael J. Devine, Mark Coles, Christopher D. Buckley, Susanna J. Dunachie, Alexander J. Clarke

**Affiliations:** 1 Kennedy Institute of Rheumatology, University of Oxford, Oxford, UK; 2 Nuffield Department of Medicine Centre for Global Health Research, Nuffield Department of Clinical Medicine, University of Oxford, Oxford, UK; 3 Mahidol-Oxford Tropical Medicine Research Unit, Mahidol University, Bangkok, Thailand; 4 Metabolomics STP, The Francis Crick Institute, London NW1 1AT, UK; 5 Mitochondrial Neurobiology Laboratory, The Francis Crick Institute, London NW1 1AT, UK; Department of Clinical and Movement Neurosciences, UCL Queen Square Institute of Neurology, University College London, London WC1N 3BG, UK; 6 National Institute for Health and Care Research Oxford Biomedical Research Centre, Oxford University Hospitals NHS Foundation Trust, Oxford, UK; 7 Sir William Dunn School of Pathology, University of Oxford, UK; 8 Institute of Immunity and Transplantation, Division of Infection and Immunity, University College London

## Abstract

The rapid proliferation of germinal centre (GC) B cells requires metabolic reprogramming to meet energy demands, yet these metabolic processes are poorly understood. By integrating metabolomic and transcriptomic profiling of GC B cells, we identified that asparagine (Asn) metabolism was highly upregulated and essential for B cell function. Asparagine synthetase (ASNS) was upregulated following B cell activation through the integrated stress response sensor general control non-derepressible 2 (GCN2). Conditional deletion of *Asns* in B cells impaired survival and proliferation in low Asn conditions. Removal of environmental Asn by asparaginase or dietary restriction compromised the GC reaction, impairing affinity maturation and the humoral response to influenza infection. Furthermore, metabolic adaptation to the absence of Asn required ASNS, and oxidative phosphorylation, mitochondrial homeostasis, and synthesis of nucleotides were particularly sensitive to Asn deprivation. These findings demonstrate that Asn metabolism acts as a key regulator of B cell function and GC homeostasis.

## Introduction

The germinal centre (GC) reaction is essential for effective humoral immunity(1). Following encounter with antigen, B cells in secondary lymphoid tissues enter the GC reaction, a cyclic process in which somatic hypermutation (SHM) in a microanatomic region known as the dark zone (DZ) leads to random mutation of immunoglobulin genes. B cells then compete to interact with and receive help from T follicular helper (T_FH_) cells in the microanatomic light zone (LZ). This process continues as affinity maturation occurs and memory B cells or plasma cells are generated.

GC B cells have some of the highest proliferation rates of all mammalian cells, yet their metabolism is unusual and incompletely understood(2). Unlike most rapidly dividing immune cells, GC B cells predominantly use fatty acid oxidation and oxidative phosphorylation (OXPHOS) rather than glycolysis(3–7), despite residing in a hypoxic and poorly vascularised micro-environment(8). The key metabolic pathways that are important in their homeostasis have not been fully defined.

Amino acid availability is a critical regulator of T cell responses, demonstrated either by alteration of environmental abundance, synthesis, or interference with cellular import through solute transporters(9–15). Amino acids are fundamentally required for protein synthesis, but are central to other metabolic processes(16), and it is apparent in T cells that availability of specific amino acids can have profound effects on their homeostasis. One such example is asparagine (Asn), a non-essential amino acid which can be synthesised from aspartate (Asp) by the enzyme asparagine synthetase (ASNS). ASNS synthesises Asn from Asp in an ATP-dependent reaction, and glutamic acid-oxaloacetic transaminase 2 (GOT2) synthesises Asp from the TCA cycle intermediate oxaloacetate(17). In CD8^+^ T cells ASNS is dynamically regulated during differentiation, and deprivation of Asn during their in vitro differentiation may either impair or augment proliferation and effector function depending on the time window in which it occurs(12–15).

In contrast, although it is known that B cell-derived lymphomas are sensitive to amino acid deprivation(18–21), and whilst loss of the capacity to synthesise serine, or deletion of CD98, a component of large neutral amino acid transporters, impairs GC formation, the importance of amino acid availability more broadly in B cell metabolism and humoral immunity is poorly understood(22).

Here, we find that GC B cells have high levels of amino acid uptake and protein synthesis *in vivo*. Using integrated multiomic analysis we identify asparagine (Asn), a non-essential amino acid, as a critical regulator of B cell homeostasis and GC maintenance. Mice with conditional deletion of *Asns* in B cells have impairment of GC formation upon depletion of environmental Asn, compromising humoral immunity. ASNS is regulated by sensing through the integrated stress response, and whilst most Asn in B cells is obtained from the extracellular environment following activation, mechanistically, loss of ASNS leads to profound metabolic dysfunction characterised by impairment of OXPHOS and failure of nucleotide synthesis when Asn is limited, which can be rescued by nucleotide supplementation.

## Results

### GC B cells have highly active protein synthesis and asparagine metabolism

**Figure 1:**
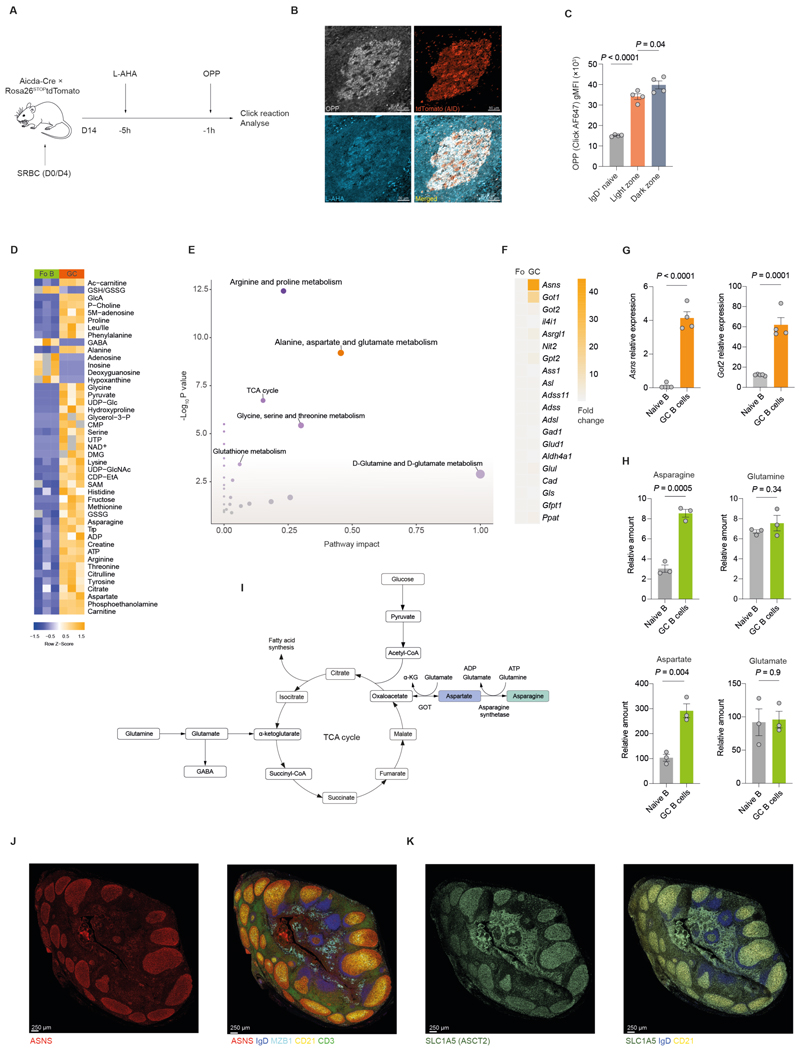
GC B cells have highly active protein synthesis and asparagine metabolism **A**. Schematic of in vivo bio-orthogonal non-canonical amino acid tagging. **B**. Representative immunofluorescence images of splenic GCs labelled with OPP, tdTomato (AID), and L-AHA. Scale bar: 50μm. **C**. Quantification of OPP (20µM) incorporation ex vivo (30 min) in naïve, LZ GC and DZ GC B cells from non-reporter WT mice (n=4). **D**. Heatmap of significantly differentially abundant metabolites (P_adj_ <0.05) measured by LC-MS (n=3 pools of 3 mice/pool). **E**. Integrated pathway analysis of differentially abundant metabolites from **F** and expressed genes from ImmGen in GC compared to naïve B cells (GSE15907). **F**. Heatmap of relative gene expression of KEGG Alanine, Aspartate, and Glutamate pathway in GC vs naïve B cells, represented as fold change. Data are from GSE133971(26). **G**. Relative expression of *Asns* and *Got2* in GC and naïve B cells at day 7 post SRBC immunization, normalised to *Ubiquitin C* (*Ubc*) (n=4 mice). **H**. Relative amounts of asparagine, aspartate, glutamine, and glutamate measured by LC-MS (n=3 pools of 3 mice) as in **F**. **I**. Diagram of asparagine synthesis and its association with the TCA cycle. **J**. Multiplexed CellDIVE images of normal human tonsil section, with immunofluorescence staining for ASNS, CD21, IgD, MZB1 and CD3. Representative of 3 separate tonsils. Scale bar = 250μm. **K**. Multiplexed CellDIVE images of normal human tonsil serial section of **L**, with immunofluorescence staining for SLC1A5 (ASCT2), CD21 and IgD. Representative of 3 separate tonsils. Scale bar = 250μm. Statistical significance was determined by one-way ANOVA with Tukey’s multiple testing correction (C), two-way ANOVA with Tukey’s multiple testing correction (D), hypergeometric test (E), unpaired two-tailed t test (G, H)). Data representative of two independent experiments (B-D, G, H). Data are presented as the mean ± SEM.

To evaluate protein synthesis rates in GC B cells we examined expression of CD98, which forms heterodimers with the amino acid transporter protein SLC7A5 to make L-type amino acid transporter 1 (LAT1). CD98 was upregulated in GC B cells compared with IgD^+^ naïve B cells (fig. S1A-B), as previously reported(22). To more directly estimate protein synthesis rates in GC B cells, we used bio-orthogonal non-canonical amino acid tagging (BONCAT) with the amino acid analogues L-azidohomoalanine (L-AHA), and O-propargyl-puromycin (OPP)(23) (Fig. 1A, fig. S1C-D). We found that incorporation of L-AHA and OPP were upregulated in the GC compared with the surrounding follicle of naïve B cells (Fig. 1B). We also labelled GC B cells with OPP *ex vivo*, and also observed an increase in OPP signal in GC B cells compared with naïve IgD^+^ B cells (Fig. 1C). Within the GC B cell population, signal was highest in the dark zone (CXCR4^hi^ CD86^low^) (Fig. 1C, fig. S1E). These results suggest that GC B cells have high rates of both amino acid transporter expression and protein synthesis.

We next directly profiled the metabolite content of GC B cells using liquid chromatography-mass spectrometry (LC-MS). This revealed a broad increase in the levels of metabolites in GC B cells, with overrepresentation of intermediates of glycolysis, the TCA cycle, and most amino acids (Fig. 1D). Notably however, nucleotide precursor molecules (e.g.adenosine and hypoxanthine) were depleted in GC B cells. To refine our approach, we fused metabolite and transcriptional datasets(24) and performed integrated pathway analysis on the combined data using hypergeometric set testing(25) (Fig. 1E). We found that there was enrichment of amino acid metabolic pathways in GC B cells, most notably the Kyoto Encyclopedia of Genes and Genomes (KEGG) Alanine, Aspartate, and Glutamate (AAG) metabolism pathway. Examination of the leading-edge genes of the AAG pathway in an independent transcriptional dataset revealed an approximately 40-fold increase in the expression of asparagine synthetase (*Asns*) and a 15-fold increase in glutamic oxaloacetic transaminase-2 (*Got2*) in GC B cells compared with naïve follicular B cells(26) (Fig. 1F). We confirmed upregulation of *Asns* and *Got2* in sorted GC B cells by qPCR (Fig. 1G) and by immunoblotting (fig. S1F). The relative levels of both Asn and Asp were increased in GC B cells compared with naïve B cells, which had equivalent glutamate (Glu) and glutamine (Gln) levels (Fig. 1H).

To examine expression of ASNS, the enzyme which synthesises Asn from Asp (Fig. 1I) in its spatial context, we performed multiplex imaging of human tonsils (Fig. 1J and fig. S1G). ASNS was strongly expressed in GCs and MZB1^+^ plasmablasts. There was negligible expression in the IgD^+^ B cell follicle or in the CD3^+^ T cell zone (fig. S1H). ASNS levels were spatially regulated within the GC, with higher expression in the DZ, where proliferation and SHM occurs (fig. S1H). We also performed immunostaining for the amino acid transporter Alanine serine cysteine transporter 2 (ASCT2), the main transporter of Asn and which also transports L-AHA(13, 27), and found high expression in GC B cells, more pronounced in the DZ like ASNS (Fig. 1K and fig. S1I). These findings demonstrate that Asn metabolism is one of the most upregulated pathways in GC B cells and plasmablasts, suggesting that it may play an important role in their physiology.

### B cells require ASNS when availability of Asn is limited

**Figure 2:**
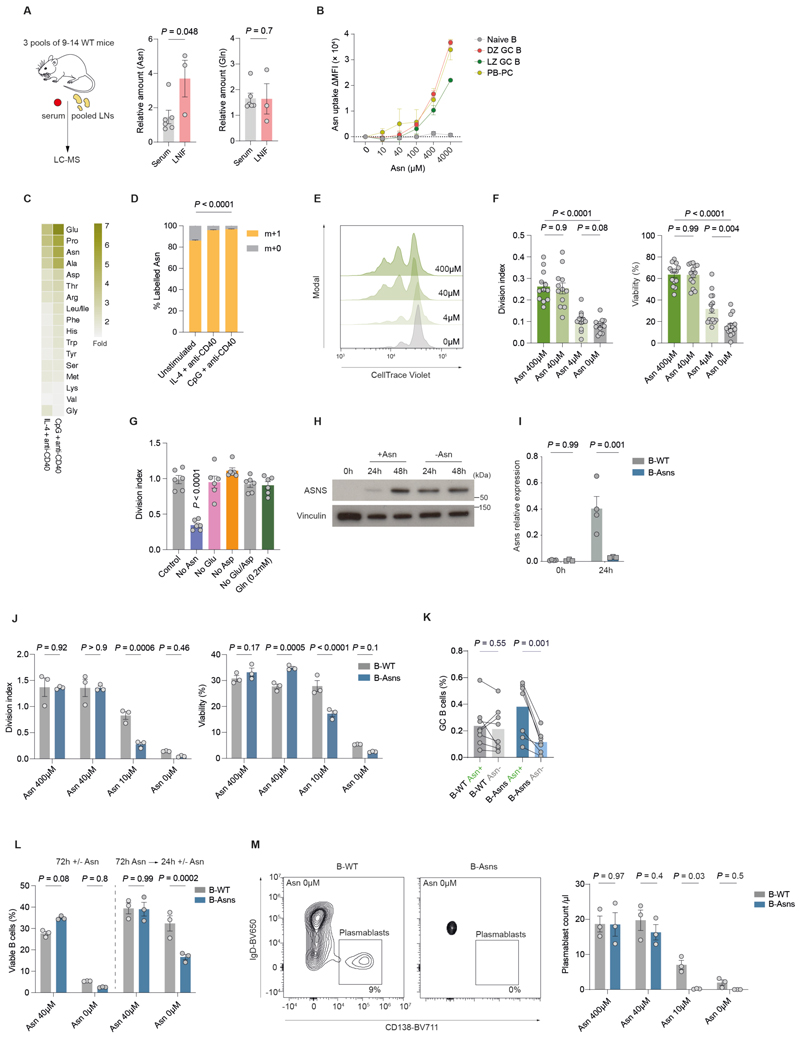
B cells require ASNS when the availability of Asn is limited **A**. Relative amounts of Asn and Gln in serum and lymph node interstitial fluid (LNIF) measured by LC-MS (n=3 pools of 9-14 WT mice for LNIF and 2-3 WT mice for serum). **B**. Quantification of HPG ΔMFI as in **fig. S2A**. **C**. Heatmap of fold change of indicated amino acids in B cells after 24h (n=3 mice). **D**. Fractional labelling following culture with ^15^N_1_-Asn for 24h (n=3 mice). **E**. Representative histogram of CellTrace Violet in B cells stimulated with IL-4 and anti-CD40 and cultured for 72h with the indicated concentration of Asn. **F**. Division index and viability of B cells cultured as in **E**. Each dot represents a single mouse. **G**. Division index of B cells stimulated as in **E** in the presence or absence of the indicated amino acids (n=6 mice **H**. Immunoblot of ASNS in B cells stimulated with IL-4 and anti-CD40 with or without Asn, at the indicated timepoints. **I**. Relative expression of *Asns* by qPCR in stimulated (IL-4 and anti-CD40 for 24h) B cells (n=4 mice per group). **J**. Division index and viability of B cells from B-Asns and B-WT mice stimulated for 72h with IL-4 and anti-CD40 at the indicated concentration of Asn (n=3 mice per group). **K**. Quantification of GC B cell proportions by flow cytometry in lymph node slices. Each dot represents single lymph node (n=8 lymph nodes from n=4 mice). **L**. Viability of B cells stimulated as in **E** (left panel), or prestimulated in the presence of Asn for 72h before Asn withdrawal (right panel)(n=3 mice). **M**. Representative flow cytometry plots and quantification of plasmablasts generated following stimulation of B cells with LPS and IL-4 for 72h. Statistical significance was determined by a two-tailed Mann–Whitney U-test (A), repeated measures one way ANOVA with Tukey’s multiple testing correction (F-G) and ordinary one way ANOVA with Dunnet’s multiple testing correction (E,H), paired two-tailed t test (N) or two-way ANOVA with Šidák’s multiple testing correction (J-L,O,Q). Data are pooled from two (J-L) or three (A,F,G,M) independent experiments or representative of two independent experiments (B-D, H). Data are presented as the mean ± SEM.

To approximate the concentration of Asn and Gln encountered by B cells in secondary lymphoid tissue, we eluted interstitial fluid from pooled lymph nodes (LNIF) of wild type mice, and compared this to serum. We found that Asn levels in LNIF were substantially higher than those in serum, whilst Gln levels were comparable (Fig. 2A).

Asn is a non-essential amino acid and can be either acquired exogenously or synthesised by ASNS. To understand these relative contributions to Asn metabolism, we first functionally evaluated Asn uptake in ex vivo GC B cells, using an indirect assay in which the bio-orthogonal amino acid analogue homopropargylglycine (HPG) competes for transport through ASCT2 with Asn. HPG is then fluorescently labelled using Click chemistry, and its uptake measured by flow cytometry(27). We found that there was clear dose-dependent competition between HPG and Asn in GC B cells and plasmablasts (fig. S2A). This was most marked in DZ GC B cells, in keeping with their high levels of OPP incorporation and ASNS expression, and indicating substantially elevated Asn uptake compared to naïve B cells (Fig. 2B).

To understand the dynamics of Asn uptake following B cell activation, we then stimulated wild type B cells in vitro with either the TLR9 agonist CpG and agonistic anti-CD40 antibody, or IL-4 and agonistic anti-CD40, for 24h in the presence of ^15^N-labelled Asn, and quantified amino acids by mass spectrometry. We found a generalised increase in the intracellular concentrations of most amino acids, but in particular proline (Pro), glutamate (Glu), and Asn (Fig. 2C). The majority of Asn was labelled with ^15^N in all conditions, indicating high exogenous uptake, and in preference to endogenous synthesis, which is bioenergetically demanding (Fig. 2D).

Next, to determine the requirements of B cells for exogenous Asn, we stimulated them in the presence of varying concentrations of Asn. Standard RPMI-1640 contains higher concentrations of Asn (378μM) compared with those found in plasma (~40μM) and cerebrospinal fluid (CSF) (~4μM)(28, 29). When B cells were deprived of Asn or supplemented with a low concentration of Asn (4μM), and stimulated with IL-4 and anti-CD40 for 72h, there was a severe reduction in cell proliferation and viability, compared with supplementation of Asn at 40μM or 400μM (Fig. 2E-F). We noted no decrease in cell proliferation or viability on withdrawal of exogenous aspartate (Asp) and/or Glu (Fig. 2G and fig. S2B). There was a reduction in the expression of the activation markers CD86 and MHC-II following Asn deprivation, nascent protein synthesis measured by OPP incorporation was lower at 24h (fig. S2C-E), and apoptosis was increased (fig. S2F). ASNS levels in B cells increased substantially over time following stimulation, and the absence of Asn led to higher ASNS expression at 24h (Fig. 2H).

To determine the role of Asn synthesis in B cell homeostasis, we generated *Cd79a*-*Cre* × *Asns* ^LoxP^ mice (B-Asns hereafter), in which Cre recombinase is expressed at earliest stages of B cell development(30). There was no evident B cell developmental defect, and the mature B cell compartment was normal, with effective deletion of *Asns*. (Fig. 2I, fig. S2G-I). When Asn was restricted in culture, survival and proliferation were severely compromised in B-WT B cells, and to a greater extent in B-Asns B cells, but were normal under Asn-replete conditions (40µM and 400µM) (Fig. 2J). We next used a live ex vivo lymph node slice platform to investigate how Asn deprivation affected GC B cells (fig. S2J) (31). We found that GC B cells from B-Asns lymph nodes were reduced after 20h of culture without Asn, but total B cell proportions were unaffected (Fig. 2K, fig. S2K). Since GC B cells express ASNS, and B cells upregulate ASNS following stimulation, we reasoned that it conferred protection against Asn deprivation. We therefore pre-stimulated B-Asns or B-WT B cells in the presence of Asn, which was then withdrawn for 24 hours. In contrast to when Asn was initially absent, B-WT B cells were minimally affected by the absence of Asn (Fig. 2L). However, in B-Asns B cells this protective effect was attenuated. Plasmablast differentiation induced by LPS and IL-4 was largely abolished in B-Asns B cells when Asn was absent or at low concentrations (10μM), but remained unchanged in the presence of Asn (Fig. 2M, fig. S2L).

These findings indicate Asn uptake and synthesis is specifically required during initial B cell activation, and loss of synthetic capacity leads to severely compromised cell division and survival in B cells in low Asn conditions.

### The GC reaction is sensitive to Asn deprivation

**Figure 3:**
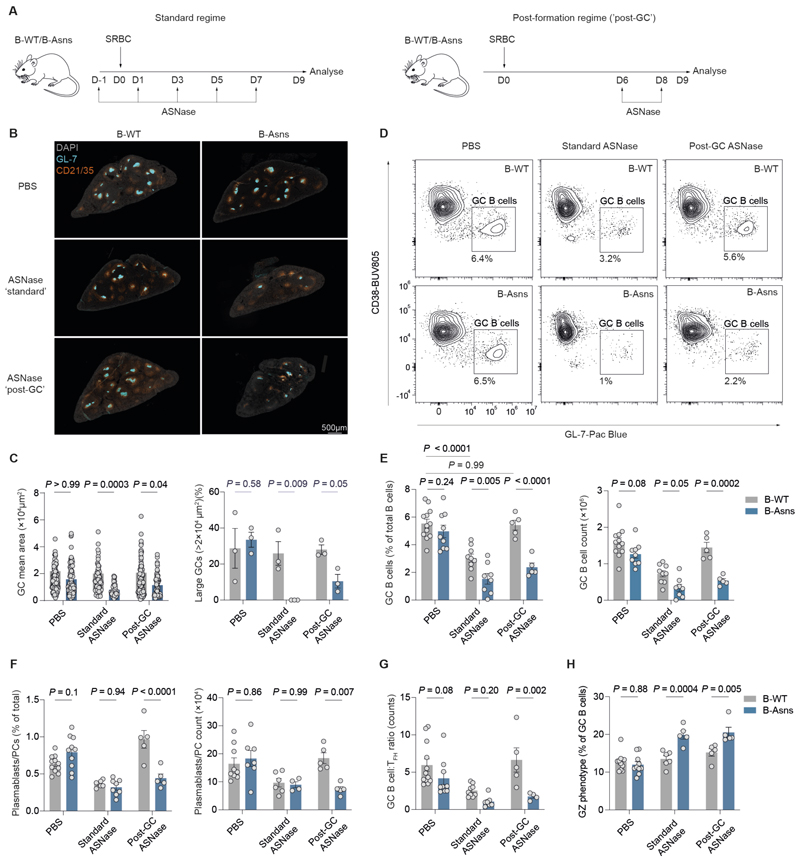
The GC reaction is sensitive to Asn deprivation **A**. Schematic of in vivo ASNase administration regimes. **B**. Immunohistochemistry of representative spleen sections (8μm) at day 9 post SRBC immunisation. GL-7 and CD21/35 highlights GCs and B cell follicles, respectively. DAPI is used as background staining. Scale bar 500μm. **C**. Image quantification of splenic GCs from **B. Left panel**: mean area of individual GCs (μm^2^). Each data point represent a GC pooled from n=3 mice per condition. **Right panel**: frequency of GCs larger than 20000µm^2^ (0.02mm^2^). Each data point indicates a mouse. n=3 from each condition. **D**. Representative flow cytometry plot of GC B cells (CD19^+^CD38^-^GL-7^+^) at day 9 post-immunisation with SRBC, from B-WT and B-Asns mice treated with PBS, standard ASNase or post-GC ASNase. **E**. Quantification of splenic CD38^-^GL-7^+^ GC B cell proportions (% of CD4^-^CD19^+^ B cells) and absolute counts. Each data point represents a single mouse. **F**. Quantification of IgD^-^IRF4^+^CD138^+^ splenic plasmablast/plasma cell proportions (% of total) and absolute counts. Each data point represents a single mouse. **G**. Quantification of the GC B cell numbers relative to T_FH_ numbers gated as CXCR5^hi^ PD-1^+^ within CD19^-^ CD4^+^ FoxP3^-^ T cells. Each data point represents a single mouse. **H**. Quantification of the proportion of the grey zone (GZ) (CD86^hi^CXCR4^hi^) GC B cells (as % of GC B cells). Statistical significance was determined by two-way ANOVA with Šidák’s multiple testing correction. Data are pooled from ≥3 independent experiments for each panel. Data are presented as the mean ± SEM.

To understand the effect of Asn deprivation on the GC reaction *in vivo*, we treated B-Asns and B-WT mice with asparaginase (ASNase), an enzyme which hydrolyses Asn (32). We used two treatment schedules (Fig. 3A). In the ‘standard’ schedule, we administered ASNase from one day prior to immunisation with SRBC, and then every two days over a nine day period before analysis. This includes the period of GC establishment and expansion. In the ‘post-formation’ schedule, we used only two doses, at days six and eight following immunisation, therefore targeting established GCs. ASNase effectively depleted Asn without affecting Gln in the serum, and minimally affected naïve B cells, marginal zone B cells, and T follicular helper (T_FH_) and T follicular regulatory (T_FR_) (fig. S3A-G).

We found that following ASNase administration, the GC reaction was compromised in B-Asns mice with both regimes, with reduced GC B cell and plasmablast proportions, numbers, and GC area (Fig. 3B-F, fig. S3H-I). B-WT mice however, were insensitive to the post-formation regime. The effect of genotype on plasmablast numbers was only evident with later ASNase treatment. There was a severe reduction in GC B cells relative to T_FH_ numbers in B-Asns mice treated with the post-GC formation regime, suggesting a selective impact on GC B cells (Fig. 3G).

We then measured the effect of post-GC formation ASNase on the humoral response to the protein-hapten conjugate immunogen NP-CGG (fig. S4A-B). This ASNase regime reduced the levels of both low- and high-affinity antibodies (NP_20_ and NP_2_, respectively), with minimal genotype effect (fig. S4C).

Within the GC B cell compartment, there was a reduction in DZ phenotype cells in treated B-Asns mice, but an increase in those with a ‘grey zone’ (GZ) phenotype (Fig. 3H and fig. S4D-F). The GZ is the compartment of peak cell proliferation in the GC, and is characterised by high expression of the cell cycle protein cyclin B1, and of cells in G2/M phase(33)(fig. S4G). We did not detect a defect in S phase in GC B cells, as measured by incorporation of the thymidine analogue EdU *in vivo*, which was slightly increased following ASNase treatment (fig. S4H-I).

Together these results suggest that deprivation of Asn with ASNase compromises the GC reaction in the absence of ASNS, possibly impairing plasmablast formation.

### Asn is required for GC B cell function

To further examine the requirement for Asn in B cells, we next used dietary limitation of Asn. We generated mixed chimeras with CD45.1 wild type and CD45.2 B-Asns or B-WT bone marrow, then fed an Asn-free or normal diet for 12 days (fig, S5A).

**Figure 4:**
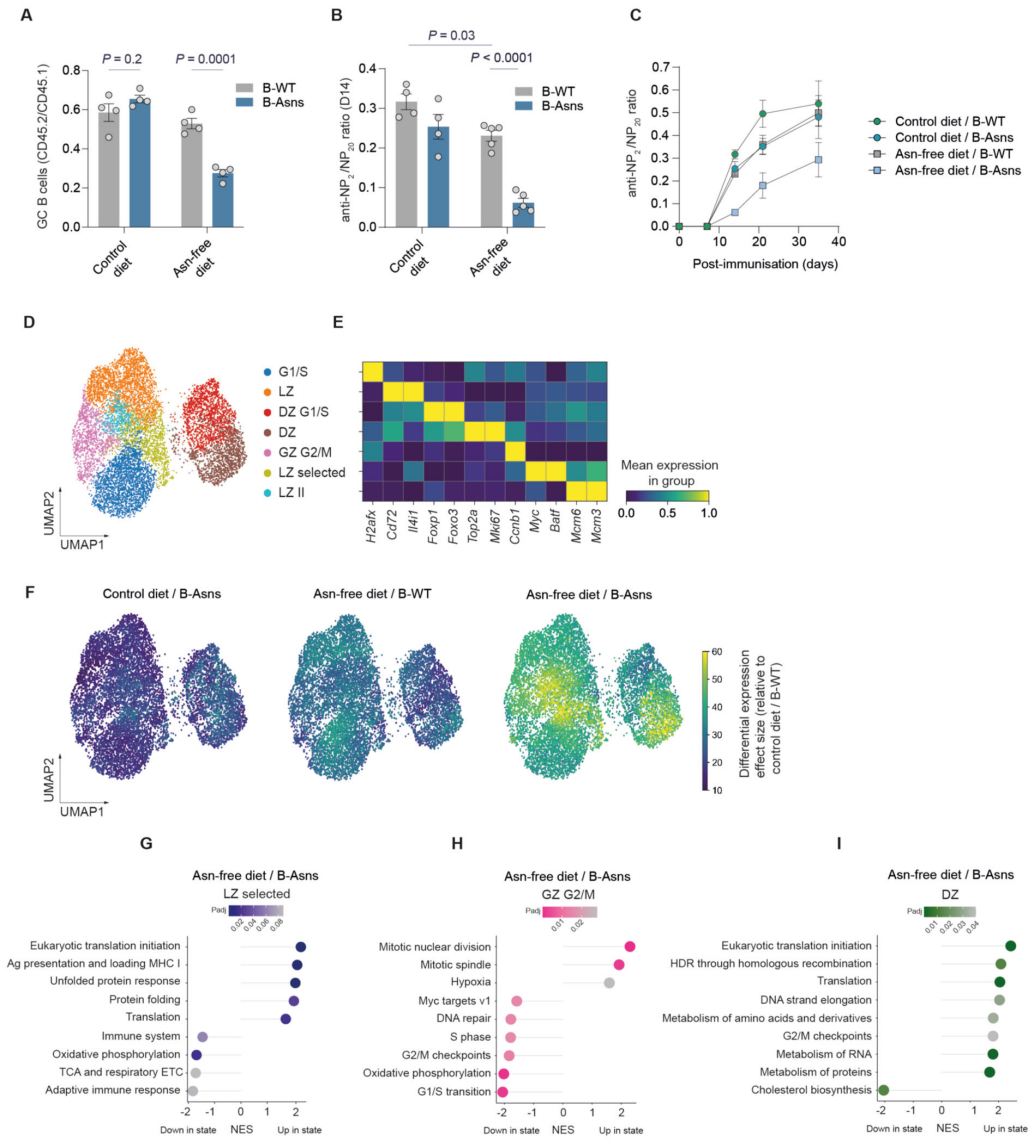
Asn is required for GC B cell function **A**. Ratio of splenic GC B cells in CD45.2^+^ B cells normalised to CD45.1^+^ WT counterparts from bone marrow chimeric mice. Each data point represents a single mouse. **B**. Quantification of NP_2_:NP_20_ ratio (at 1:200 dilution) of IgG1 anti-NP antibodies at day 14, n=4-5 mice each condition. **C**. Comparison of the ratio of IgG1 NP-specific high-affinity antibodies to low-affinity antibodies detected by binding to NP_2_ and NP_>20_ antigens, respectively, from B-WT/control diet (n = 4), B-Asns/control diet (n = 4), B-WT/Asn-free diet (n = 5), B-Asns/Asn-free diet (n=5) across different time points. **D**. UMAP and cluster annotation based on multiresolution variational inference (MrVI) latent space of integrated control diet/B-WT (n=2550 cells), control diet/B-Asns (n=2971 cells), Asn-free diet/B-WT (n=1944 cells), and Asn-free diet/B-Asns (n=2700 cells) (n = 3 mice per condition). **E**. Heatmap of selected differentially expressed genes used to identify clusters, as in **D**. **F**. Multiresolution variational inference (MrVI) cluster-free differential gene expression effect size for the indicated conditions relative to the control diet/B-WT group. **G**. Gene set enrichment analysis (GSEA) of the indicated pathways in the ‘LZ selected’ cluster of Asn-free diet/B-Asns relative to control diet/B-WT groups. **H**. GSEA of the indicated pathways in the in the ‘GZ G2/M’ cluster of Asn-free diet/B-Asns relative to control diet/B-WT groups. **I**. GSEA of the indicated pathways in the in the ‘DZ’ cluster of Asn-free diet/B-Asns relative to control diet/B-WT groups. Statistical significance was determined by two-way ANOVA with Tukey’s multiple testing correction (D) or Šidák’s multiple testing correction (B) and adaptive multi-level split Monte-Carlo scheme (I-K). Data are pooled from two independent experiments (A-C). Data are presented as the mean ± SEM.

The relative proportion of GC B cells in CD45.2 B-Asns B cells was lower in mice fed an Asn-free diet, and the DZ/LZ ratio was reversed compared to B-WT cells (Fig. 4A, fig. S5B), which reproduced our findings with ASNase. Splenic plasmablasts of B-Asns origin were also reduced (fig. S5C). We found a reduction in the frequency of SRBC-binding B-Asns-derived GC B cells in mice fed an Asn-free diet (fig. S5D-E), suggesting impaired development of antigen-binding clones.

We then examined the effect of dietary Asn deprivation on the humoral response to NP-CGG in a non-chimeric setting, in B-Asns and B-WT mice (fig. S5F).

At day 14 post immunization, levels of both low- and high-affinity anti-NP IgG1 were reduced in sera, also seen in B-WT mice given an Asn-free diet, which was maintained over time (Fig. 4B-C, fig. S5G-H). We did not observe a large difference in anti-NP IgM, which is of likely extrafollicular origin (fig. S5I). We also examined the anti-NP IgM antibody response at day 7 after immunisation with the T-independent antigen NP-Ficoll(34) (fig. S5J). There was a reduction in IgM anti-NP_20_ antibody levels in B-Asns mice on an Asn-free diet, compared to B-WT given a control diet (fig. S5K).

Forty days following immunisation with NP-CGG, we found that there was a substantial decrease in the NP-binding memory B cell compartment (fig. S5L-M). We also performed experiments in which mice were immunised with NP-CCG a week prior to dietary modification, and then at day 36 boosted with further NP-CGG (fig. S6A-B). This showed attenuation of the boost-associated increase in high-affinity anti-NP antibody levels at day 49 in B-Asns mice given an Asn-free diet (fig. S6C-D). Serum Asn levels remained unchanged on days 12 and 40 of dietary intervention, likely due to systemic compensation (fig. S6E).

We next performed scRNAseq in GC B cells from mice fed a control or Asn-free diet. We distinguished 7 clusters, with varying light zone, dark zone, and grey zone marker gene expression (Fig. 4D-E). Examining differential gene expression across all cells with a multiresolution variational inference model, we found a gradient across conditions relative to B-WT mice on control diet, with the largest effect size in B-Asns mice given an Asn-free diet (Fig. 4F). When cell state differential abundance was analysed, there were differences in all conditions compared to B-WT mice on control diet, centred on the dark zone (fig. S6F).

We identified that the LZ-selected cluster, which expressed markers of GC B cell selection such as *Myc* and *Batf*, showed the largest differential gene expression effect. We found that in this cluster under unperturbed conditions, *Asns, Slc7a5*, and *Slc3a2* were all significantly upregulated compared to other clusters, suggesting that these genes may be linked to GC B cell selection and could play a role in metabolic refuelling (fig. S6G). We analysed a bulk RNAseq dataset from the DEC205-OVA system (GSE132881 (35, 36). In this dataset, we observed that unlike their DEC205^-/-^ counterparts, DEC205^+/+^ selected LZ GC B cells upregulated several key enzymes of the AAG pathway, including *Asns* and *Got2* in direct proportion to antigen capture, which facilitates T_FH_ help (fig. S6H). Importantly, this trend mirrored the expression of *Myc* and *Batf*. Amino acid transporters *Slc7a5* and *Slc3a2* also showed similar upregulation, consistent with our scRNAseq findings in the ‘LZ selected cluster’. Using in vitro stimulated B cells, we also found that ASNS was strongly upregulated by synergistic signalling through CD40 and IL-4, mimicking T_FH_ help, supporting the transcriptional findings (fig. S7A).

We next performed gene set enrichment analysis (GSEA) in our single cell RNA sequencing dataset, using a conventional pseudobulk differential gene expression approach. Focusing on B-Asns mice given an Asn-free diet and examining clusters which showed the largest differential gene expression effect, we found that in the LZ-selected cluster, there was downregulation of OXPHOS and TCA cycle sets, and upregulation of unfolded protein response gene sets (Fig. 4G). We next examined the GZ G2/M cluster, given our findings in B-Asns mice treated with ASNase (Fig. 4H). We found increased expression of mitosis gene sets, and downregulation of G2/M checkpoint, G1/S transition, and S phase gene sets, as well as OXPHOS. In the DZ cluster there was upregulation of cell cycle-related gene sets and downregulation of cholesterol synthesis (Fig. 4I). Examining data from the mixed bone marrow chimera experiment (fig. S6A), we identified defective mitosis within the dark zone of B-Asns GC B cells under dietary Asn restriction (fig. S7B-C).

Having shown that Asn deprivation leads to compromised GC output, we then asked if supplementation with exogenous Asn would enhance it. We did not observe an increase in antibody concentration or affinity maturation (fig. S7D-E). This suggested that the amount of Asn provided by a standard diet was fully sufficient for the GC reaction over this timescale.

These data demonstrate that Asn synthesis in B cells by ASNS acts to maintain GC B cell function in the face of dietary Asn deprivation.

### Asn metabolism controls the humoral response to influenza infection

**Figure 5:**
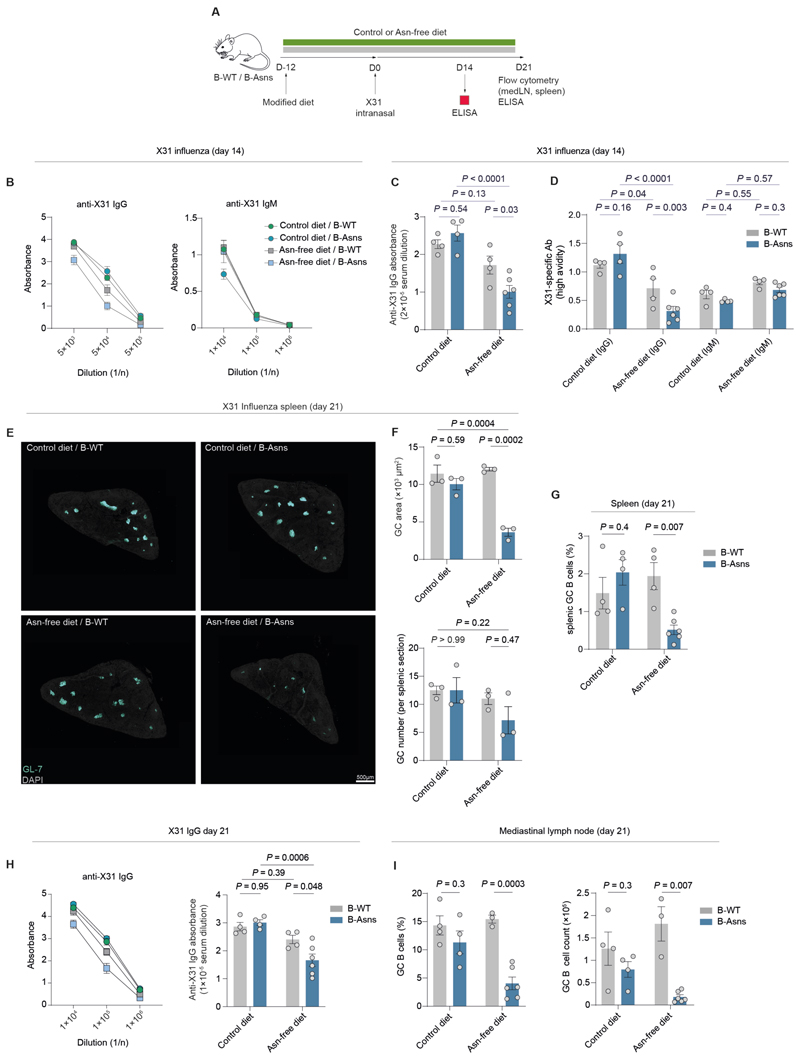
Asn metabolism controls the humoral response to influenza infection **A**. Schematic of influenza/dietary modification experiment. **B**. Dilution curves of X31-specific IgG and IgM antibodies at day 14. n=4-6 mice each condition. **C**. Comparison of X31-specific IgG (at 1/5×10^4^ dilution) at day 14. n=4-6 mice each condition. **D**. Comparison of high-avidity X31-specific IgG and IgM (at 1/5×10^4^ dilution) quantified by values in **C and fig S7B** with matching avidity values in **fig. S7D-E** at day 14. n=4-6 mice each condition. **E**. Immunohistochemistry of representative spleen sections (8μm) at day 21 post X31 influenza infection. GL-7 highlights GCs. DAPI is used as background staining. Scale bar 500µm. **F**. Image quantification of X31-induced splenic GCs from **E**. Mean GC area (μm^2^) per mouse and GC count per splenic section (average of two non-serially sliced) per mouse. Each data point indicates a mouse. n=3 from each condition. **G**. Flow cytometric quantification of splenic GC B cell (as gated in **fig. S7G**) proportions at day 21. n=4-6 mice each condition **H**. Dilution curves and quantification of X31-specific IgG antibodies at day 21. n=4-6 mice each condition as in **B**. Data pooled from two independent experiments. **I**. Quantification of GC B cell ratio and counts in mediastinal lymph nodes at day 21. n=3-6 mice each condition. Statistical significance was determined by two-way ANOVA with Tukey’s multiple testing correction (C,D,F,H) or Šidák’s multiple testing correction (G,I). Data are pooled from two independent experiments (A-I). Data are presented as the mean ± SEM.

The GC reaction is integral to humoral immune responses following flu infection. Therefore, we investigated whether Asn metabolism could affect influenza-induced GC and antibody generation (Fig. 5A). Starting from day 3 post-infection, characteristic weight loss was observed, peaking around day 7 (fig. S8A). We found that the rate of weight recovery under all conditions remained comparable, suggesting that there was no important defect in the non-B cell compartment of experimental mice (fig. S8A).

We initially examined the antibody response to X31 influenza in mouse sera. At day 14 post-infection, levels of anti-X31 IgG antibodies were substantially lower in B-Asns mice fed an Asn-free diet (Fig. 5B-C). However, we did not observe a significant difference in anti-X31 IgM levels (Fig. 5B, fig. S8B). We observed that anti-X31 IgG antibodies from B-Asns mice fed an Asn-free diet were significantly more sensitive to urea treatment, suggesting markedly impaired affinity (Fig. 5D, fig. S8C-D)(37). As expected, anti-X31 IgM antibodies exhibited greater sensitivity to urea, to a similar extent across all experimental groups, indicating lower avidity compared to anti-X31 IgG antibodies, owing to the lack of affinity maturation in their extrafollicular origin (Fig. 5D, fig. S8E). Influenza infection induces GCs in the spleen and mediastinal lymph nodes in mice(38). At day 21, we noted a severe reduction in splenic GC size, GC B cell proportions, and GC B cell counts relative to T_FH_ in B-Asns mice deprived of Asn (Fig. 5E-G, fig. S8F). Levels of anti-X31 IgG antibodies remained significantly lower in B-Asns mice fed an Asn-free diet, reproducing the findings at day 14 (Fig. 5H). Moreover, we found a robust numerical and proportional defect in mediastinal GC B cells in B-Asns mice on an Asn-free diet (Fig. 5I, fig. S8G). Additionally, there was a reduction in both the DZ to LZ and GC B to T_FH_ cell ratios, resembling the phenotype observed with ASNase treatment, but a non-significant change in mediastinal lymph node plasma cell numbers despite a decreasing trend (fig. S8H-J).

These findings indicate that Asn metabolism regulates GC-derived humoral immune responses during influenza infection, and *Asns* plays an indispensable role in this process when Asn availability is restricted.

### Disruption of Asn availability alters B cell metabolism

To understand how Asn withdrawal affects B cell metabolism, we performed stable isotope resolved LC-MS on B cells from B-WT or B-Asns mice (fig. S9A).

**Figure 6:**
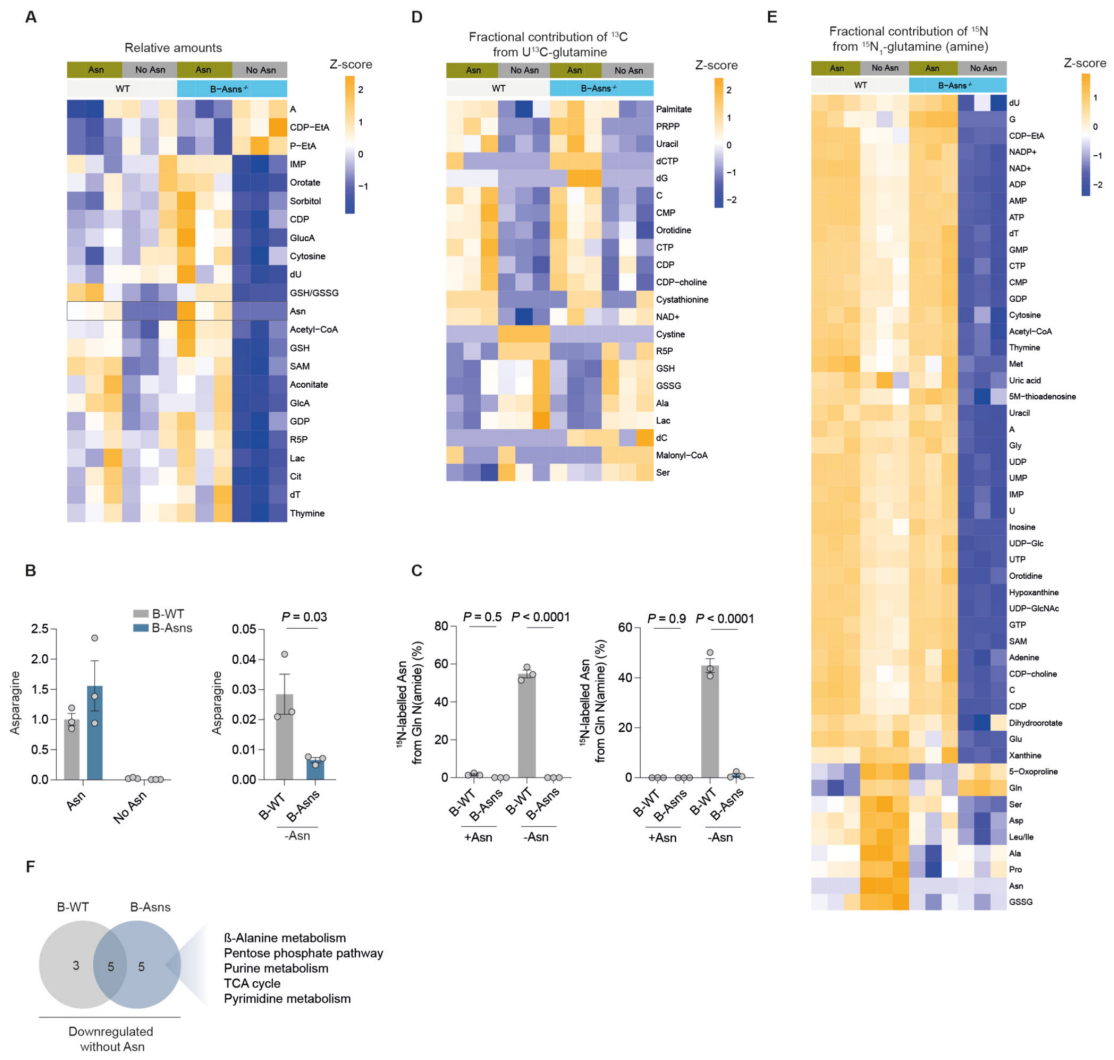
Disruption of asparagine availability alters B cell metabolism **A**. Heatmap of total relative amounts of significantly different metabolites in B cells from B-WT or B-Asns mice as in **A**. Scale represents row Z-score. Significantly differentially abundant metabolites (P_adj_ < 0.05) are shown. Representative of two independent experiments. **B**. Abundance of intracellular Asn in B cells cultured as in **A**, and rescaled plot of Asn-deprived condition. Representative of two independent experiments. **C**. Fractional contribution of ^15^N to Asn pool, derived from ^15^N_1_-amino or amide Gln, following culture as described in **A**. **D**. Heatmap of fractional contribution of ^13^C to indicated metabolites, derived from U-^13^C-Gln following culture as described in **A**. Scale represents row Z-score. Significantly differentially labelled metabolites (P_adj_ < 0.05) are shown. **E**. Heatmap of fractional contribution of ^15^N to indicated metabolites, derived from ^15^N_1_-amine Gln following culture as described in **A**, with labelled compound added for final 24h of culture. Scale represents row Z-score. Significantly differentially labelled metabolites (P_adj_ < 0.05) are shown. **F**. Venn-diagram indicating number of significantly differentially-regulated pathways in B-WT and B-Asns B cells following Asn-withdrawal, based on relative metabolite abundance as in **B**. Listed pathways are those specifically downregulated in B-Asns B cells. Statistical significance was determined by two-way ANOVA with Šidák’s multiple testing correction (B,D-F), unpaired two-tailed t test (C), or hypergeometric test (G). Data are presented as the mean ± SEM.

Intracellular Asn levels were reduced in Asn-deprived conditions in both B-WT and B-Asns B cells, confirming our previous observation that exogenous uptake is dominant, but to a greater degree in those from B-Asns mice (Fig. 6B-C). B-WT cells did not engage in significant Asn synthesis when Asn was present, with negligible labelling of Asn with Gln-derived ^15^N from either the amino or amide position, despite ASNS expression (Fig 6D).

Following Asn deprivation, around half of the intracellular Asn pool was ^15^N-labelled in B-WT B cells, reflecting de novo synthesis by ASNS. As expected, B-Asns B cells were incapable of Asn synthesis and no Asn was ^15^N labelled. There was minimal labelling of Asn from ^13^C glutamine, but around a quarter of Asp was labelled, with no difference between B-WT and B-Asns B cells, suggesting very little of the Gln-derived Asp pool was diverted to Asn synthesis (Fig. 6E, fig. S9B).

In B-WT cells, Asn deprivation had subtle effects on metabolite relative abundance, but significantly reduced fractional labelling from [U-^13^C]-glutamine and ^15^N_1_-glutamine. Despite small differences in intracellular Asn, B-Asns B cells exhibited profound metabolic dysfunction upon Asn withdrawal, with large reductions in metabolite relative amounts and glutamine-derived ^15^N incorporation (Fig. 6F).

We next performed pathway analysis to systemically describe these results. Comparing B-WT and B-Asns B cells, both deprived of Asn, there were a common set of significantly different pathways (Fig. 6G), including AAG, tRNA biosynthesis, glutathione synthesis, sphingolipid metabolism and glyoxylate. We noted that B-Asns B cells, when deprived of Asn, substantially downregulated metabolites in the TCA cycle, nucleotide biosynthesis. pentose phosphate pathway, and β-alanine metabolic pathways (Fig. 6G). These findings demonstrate that Asn is essential for regulating metabolic homeostasis in B cells.

### Mitochondrial function in B cells requires Asn

**Figure 7:**
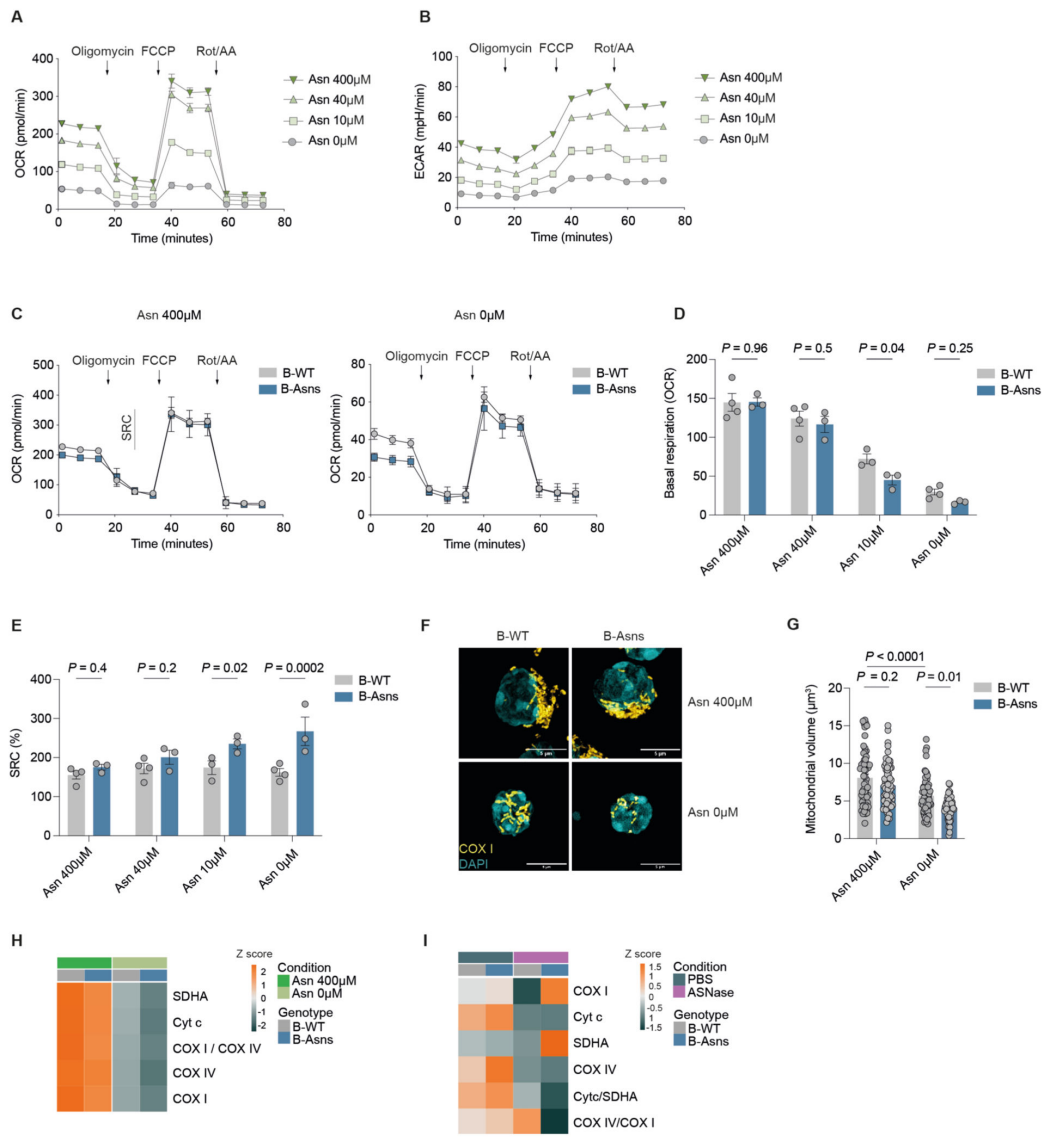
Mitochondrial function in B cells requires Asn **A**. Seahorse extracellular flux analysis of B cells (n=3-4 mice). FCCP, carbonyl cyanide-p-trifluoromethoxyphenylhydrazone; Rot/AA, rotenone/antimycin A **B**. Seahorse extracellular flux analysis of B cells as in **A** (n=3-4 mice). Extracellular acidification rate (ECAR) was measured during the MitoStress test. **C**. Seahorse OCR quantification in B-WT (n=3-4 mice) and B-Asns (n=3 mice) B cells. Spare respiratory capacity (SRC) is illustrated. **D**. Quantification of basal OCR in B-WT and B-Asns B cells. Each data point represents a mouse. **E**. Quantification of SRC (%) based on quantifications in **fig. S9B** and **Fig. 7D**. **F**. 3D lattice SIM images of COX I and DAPI in B-WT and B-Asns B. Scale bar 5µm. **G**. Quantification of mitochondrial volume as in **F**. Each data point represents a cell (n=57 B-WT/Asn400, n=66 B-Asns/Asn400, n=65 B-WT/Asn0, n=53 B-Asns/Asn0) pooled from n=3 mice B-WT or B-Asns mice. **H**. Heatmap of row z-scores for the gMFI of indicated ETC proteins (mean of n = 2, n = 3 mice per group in total). **I**. Heatmap of row z-scores for the gMFI of indicated ETC proteins and ratios (mean of n = 3-4) mice treated with ASNase (standard regime). Statistical significance was determined by two-way ANOVA with Tukey’s multiple testing correction (D-E, G). Data are representative of two (G-I) or three pooled independent experiments with 5-6 technical replicates per mouse (A-E) Data are presented as the mean ± SEM.

Given the abnormalities in TCA cycle metabolism we observed following Asn withdrawal, we next performed extracellular flux analysis to explore OXPHOS and glycolysis in a dynamic setting. We found that Asn concentration was strongly associated with oxygen consumption rate (OCR) and extracellular acidification (ECAR), reflecting OXPHOS and lactate production respectively (Fig. 7A-E, fig. S10A-B). The deleterious effect of low Asn was more pronounced in B-Asns B cells (Fig. 7C-D). In keeping with our metabolite profiling, this effect was only seen when Asn was limited. Surprisingly, the maximal respiration induced by the mitochondrial uncoupler carbonyl cyanide-p-trifluoromethoxyphenylhydrazone (FCCP) remained unchanged between B-WT and B-Asns B cells across all Asn concentrations, resulting in a significantly higher maximal-to-basal respiration ratio, known as the spare respiratory capacity (SRC), in B-Asns B cells (Fig. 7E, fig. S10B).

Supplementation with a cell-permeable analogue of the TCA cycle metabolite alpha-ketoglutarate (α-KG), dimethyl 2-oxoglutarate (DM-OG) has been reported to increase cell viability in T cells treated with L-asparaginase(39). We therefore hypothesised that supplementation with DM-OG could potentially restore defective mitochondrial metabolism and improve B cell homeostasis in Asn-deficient conditions. However, DM-OG failed to rescue the defective B cell survival in B-WT and B-Asns B cells under Asn-free conditions (fig. S10C).

We next directly imaged mitochondria in B-WT and B-Asns B cells using 3D Lattice structured illumination microscopy (SIM) (Fig. 7F). Mitochondrial volume and count were not different when Asn was present, but were reduced in the absence of Asn, and again this was seen to a greater extent in B-Asns B cells (Fig. 7G, fig. S10D).

The electron transport chain (ETC) is crucial for OXPHOS activity. B cells undergo profound mitochondrial remodelling upon activation associated with ETC protein expression(2). Using flow cytometry, we characterised the ETC protein abundances in activated B cells and found that Asn deprivation led to a substantial reduction in the expression of ETC proteins cytochrome c oxidase subunit I (COX I), cytochrome c oxidase subunit IV (COX IV), succinate dehydrogenase A (SDHA), and cytochrome c (Cyt c) in B-WT and to a greater extent in B-Asns B cells (Fig. 7H).

We next examined ETC proteins in *ex vivo* GC B cells from B-WT and B-Asns mice following ASNase. This again showed a decrease in levels of Cyt c and COX IV, but upregulation of SDHA and COX I, suggesting divergent contributions of Asn to mitochondrial regulation in GC B cells (Fig. 7I).

Taken together, these findings indicate that Asn availability maintains mitochondrial ETC homeostasis, and ASNS regulates mitochondrial respiration in low Asn conditions.

### Asn regulates nucleotide metabolism and the integrated stress response

Mitochondrial function is intimately linked to nucleotide metabolism, and in our GC B cell metabolomics experiment we found low levels of nucleotides, suggesting that their availability may be limiting, even in normal physiology (Fig. 1D)(40). When examining the effect of Asn on B cell metabolite abundance, we found that nucleotide synthesis pathways were selectively impaired in B-Asns B cells following Asn deprivation (Fig. 6F). Detailed metabolite pathway analysis revealed significant downregulation of intermediate metabolites in both de novo purine and pyrimidine biosynthesis branches (fig. S11A). Orotate and uridine monophosphate (UMP), a common precursor of pyrimidine end-products cytosine and thymidine, were downregulated in B-Asns B cells.

**Figure 8:**
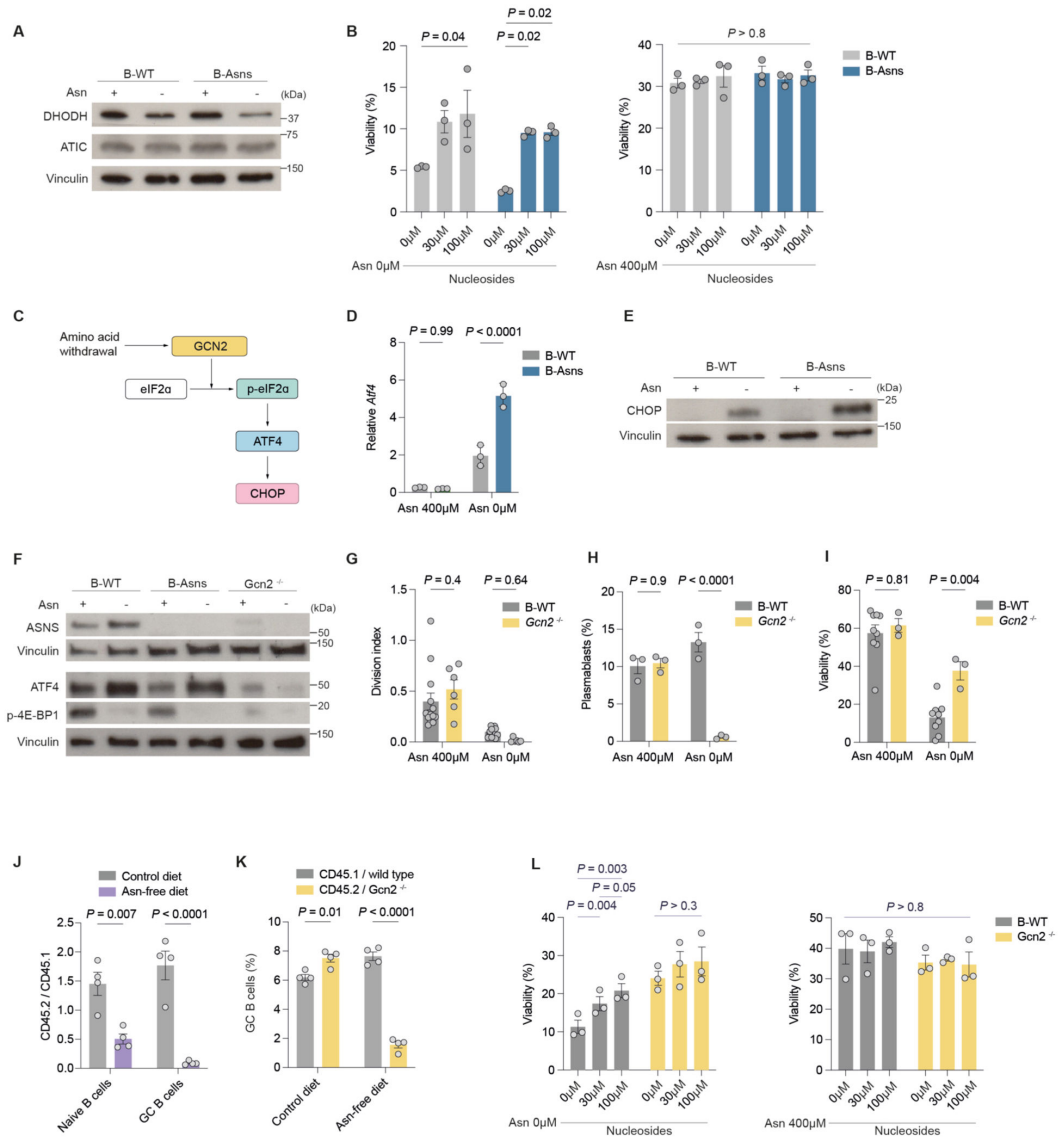
Asn regulates nucleotide metabolism and the integrated stress response **A**. Immunoblot of DHODH and ATIC in B cells stimulated for 24h. **B**. Viability of B cells stimulated for 72h, supplemented with adenosine, thymidine, uridine, cytidine, and guanosine at 0μM, 30μM or 100μM each (n=3 mice per group). **C**. Schematic of the GCN2 branch of the integrated stress response. **D**. Relative expression of *Atf4* in B cells stimulated for 72h (n=3 mice per group). **E**. Immunoblot of CHOP in B cells stimulated for 24h. **F**. Immunoblot of ASNS, ATF4, phospho-4E-BP1 in B cells stimulated for 24h. **G**. Division index of *Gcn2*^*-/-*^ and wild type B cells stimulated for 72h. Each data point represents a mouse. **H**. Quantification of plasmablast differentiation of *Gcn2*^*-/-*^ and wild type B cells following stimulation with LPS and IL-4 for 72h (n=3 mice per group). **I**. Viability of *Gcn2*^*-/-*^ and wild type B cells stimulated for 72h (n=3 *Gcn2*^*-/-*^ mice and n=9 wild type mice). **J**. CD45.2 *Gcn2*^-/-^ naïve and GC B cell abundance relative to CD45.1 wild type counterparts, fed control or Asn-free diets (n=4 mice). **K**. GC B cell frequencies in the CD45.2^+^*Gcn2*^-/-^ and CD45.1^+^ wild type B cell compartments under control or Asn-free diet (n=4 mice) settings. **L**. Viability of B cells from B-WT or *Gcn2*^*-/-*^ mice (n=3 mice) stimulated for 72h, supplemented with adenosine, thymidine, uridine, cytidine, and guanosine at 0μM, 30μM or 100μM each. Statistical significance was determined by repeated measure two-way ANOVA with Tukey’s multiple testing correction (B,D,K) or Šidák’s multiple testing correction (G-I,M), one-way ANOVA with Tukey’s multiple testing correction (L). Data are representative of two independent experiments (A, D-F) or pooled from two (B,H,J-L) or ≥3 experiments (G, I). Data are presented as the mean ± SEM.

The enzyme catalysing the oxidation of dihydroorotate to orotate is dihydroorotate dehydrogenase (DHODH), which is located in the mitochondria and forms a functional link between de novo pyrimidine biosynthesis and ETC activity(41, 42). We quantified DHODH levels and found it markedly reduced with Asn deprivation (Fig. 8A). Notably, B-Asns B cells had the lowest DHODH levels in the absence of Asn, consistent with their lower mitochondrial mass and significantly downregulated pyrimidine metabolites. Interestingly, 5-aminoimidazole-4-carboxamide ribonucleotide formyltransferase/IMP cyclohydrolase (ATIC), which is responsible for the synthesis of inosine monophosphate (IMP) in the de novo purine synthesis pathway was not altered at the protein level (Fig. 8A).

We next performed a rescue experiment by supplementing B-WT and B-Asns B cells with nucleosides including guanosine, adenosine, thymidine, cytidine, and uridine, which bypasses the activity of nucleotide biosynthetic enzymes, including DHODH. In conditions of Asn restriction, nucleoside supplementation substantially increased viability in both B-WT and B-Asns B cells following 72h culture (Fig. 8B), but was unable to restore normal proliferative capacity (fig. S11B). Furthermore, nucleoside supplementation did not affect B cell viability or proliferation when Asn was unrestricted.

We next examined the temporal dynamics of phosphorylation of the integrated stress response protein eIF2α, and found that p-eIF2α was increased at 6 hours following stimulation in the absence of Asn (Fig. 8C and fig. S11C). *Atf4* transcription was elevated with Asn deprivation, and this effect was significantly enhanced in B-Asns B cells (Fig. 8D). CHOP was highly expressed in B-Asns B cells deprived of Asn, and to a lesser extent in B-WT B cells, following in vitro activation (Fig. 8E). We observed a reduction in mTORC1 activation assessed by phosphorylation of 4E-BP1 and S6 when B-Asns B cells were stimulated without Asn (Fig. 8F, fig. S11D). This was accompanied by an increase in expression of the amino acid transporter component CD98, and SLC7A1 (fig. S11D). However, when we examined ex vivo GC B cells from mice treated with ASNase, whilst p-eIF2α was elevated following ASNase in B-Asns mice, we also observed an unexpected increase in p-4E-BP1, p-S6, and the cell cycle regulator phosphorylated-Retinoblastoma protein (p-Rb) (fig. S11E).

To directly assess the importance of the GCN2-mediated ISR in Asn synthesis, we next examined *Gcn2*^-/-^ B cells. *Gcn2*^-/-^ B cells were unable to upregulate either ATF4 or ASNS following stimulation (Fig. 8F). Stimulation of *Gcn2*^-/-^ B cells resulted in a similar phenotype to B-Asns B cells, albeit with a paradoxical increase in viability in the absence of Asn (Fig 8G-I). We then treated B cells with the GCN2 activator halofuginone(43), and found that even when Asn was present, very low concentrations led to decreased survival (fig. S11F). This suggested that the extent of the ISR was carefully balanced in activated B cells, and excessive ISR activation was harmful. The GCN2 branch of the ISR is therefore essential for ASNS expression and cellular homeostasis in B cells.

We next examined the GC reaction in *Gcn2*^-/-^ mice. At day 14 post NP-CGG immunization, there was no difference in GC B cell numbers or anti-NP antibody levels compared with WT mice (fig. S11G-I). We generated mixed bone marrow chimeric mice with CD45.1 wild type nd CD45.2 *Gcn2*^*-/-*^ cells (fig. S11J). *Gcn2*^-/-^ lymphocytes were outcompeted by their wild type counterparts under Asn deprivation (Fig. 8J-K, fig. S11K). Within the *Gcn2*^-/-^ GC B cell compartment, there was disturbance of the DZ/LZ ratio, with a decrease in the DZ proportion (fig. S11L), similar to our previous findings in B-Asns GC B cells.

Finally, we examined whether nucleotide supplementation affected survival in *Gcn2*^*-/-*^ B cells deprived of Asn. We found that unlike B-WT and B-Asns B cells, addition of nucleosides did not improve viability (Fig. 8L). This suggests that the effects seen with loss of GCN2 on B cell survival are distinct to those with ASNS alone, and nucleoside availability is not a major limiting factor in this setting.

These data show that *Asns* therefore essential to regulate B cell metabolic homeostasis, and if it cannot be obtained in sufficient amounts from the environment or synthesised by ASNS in response to activation of the ISR, there is impairment of nucleotide biosynthesis.

## Discussion

Here we show using integrated multiomics that Asn metabolism is upregulated in GC B cells and required for their homeostasis, and that deprivation of Asn either from the external environment or by loss of its synthesis strongly alters cellular metabolism, and in particular impairs OXPHOS and nucleotide synthetic capacity.

Lack of Asn severely affected cellular metabolism in B cells, and in particular OXPHOS. This was in striking contrast to reports in CD8^+^ T cells(13, 15), in which Asn deprivation leads to activation of OXPHOS and increased nucleotide synthesis, associated with enhancement of proliferative capacity and enlarged mitochondrial mass, although others have noted reduction of cell division without Asn in this cell type, and also in cancer cells(12, 14, 44). We found that Asn-deprivation led to a substantial drop in mitochondrial volume, and we observed that there was imbalance of expression of ETC proteins. It is unclear why B cells should be so much more vulnerable to the lack of Asn than CD8^+^ T cells, but may reflect more profound cellular remodelling following activation, or other differences in core metabolism. We did not observe a defect in T_FH_ cells with Asn depletion, despite their shared microenvironment with GC B cells, highlighting their divergent metabolism. However, we cannot fully exclude a more subtle functional defect.

An important consideration in all studies of metabolism *in vitro* is how to approximate the concentrations of metabolites found within tissue. Whilst this has received attention in the context of the tumour microenvironment, very little is known about normal tissues and especially within complex structures like GCs. GCs are hypoxic and poorly vascularised, but local concentrations of Asn remain unclear. We found a much higher concentration of Asn in eluted LNIF compared to serum, which was not the case for Gln. However, whether this applies in the complex microenvironment of the GC remains to be established. Advances in mass spectrometry imaging may allow more accurate quantification of metabolites in their spatial context in the future(45). Future work is also needed to validate these findings using human lymph node samples, to better understand their relevance to pathophysiology. We were also surprised to find that dietary Asn restriction remained impactful under conditions where systemic Asn levels were largely unchanged. This observation aligns with a previous report showing reduced tumour growth when dietary Asn was limited, even without changes in serum levels(44). This also underscores the potential for dietary interventions to exert sustained, localised, and tissue-specific effects, even when systemic amino acid levels appear relatively compensated. However, further research is needed to elucidate the mechanisms behind this systemic compensation.

A key finding from our work is that Asn availability acts to gate nucleotide synthesis in B cells, a role previously demonstrated in cancer cell lines(46). GC B cells have high rates of nucleic acid synthesis, in keeping with their active cell division, and our LC-MS data revealed they had low levels of nucleosides, suggestive of their consumption. Analysis of Asn synthesis from glutamine showed that even following activation, which was associated with upregulation of ASNS, the great majority of Asn was exogenously acquired. Nonetheless ASNS was essential for metabolic homeostasis and nucleotide synthesis when Asn is restricted, despite seemingly modest rates of Asn synthesis. This raises the interesting question of whether ASNS might have other, non-synthetic functions, as has been recently demonstrated for the enzyme phosphoglycerate dehydrogenase (PHGDH), whose canonical function is to synthesise serine(47). Using single cell RNA sequencing, we identified dysregulation of cell cycle gene expression pathways when Asn was deficient, and confirmed that whilst cyclin B1 was upregulated, mitosis was defective in B-Asns DZ GC B cells. Interestingly, ASNS has been found to be recruited to the mitotic spindle in dividing human cells under Asn deprivation although its function is unknown(48). We found that *Asns* and other AAG pathway genes were upregulated in *Myc*-expressing GC B cells in our scRNAseq data, also seen in the DEC-OVA model of GC B cell selection(35, 36). This suggests that activation of Asn metabolism may be involved in GC B cell selection, and the precise role of ASNS in GC B selection warrant further investigation.

The relationship between amino acid availability and nucleotide synthesis has been previously defined through mTORC1 signalling, acting via phosphorylation of the enzyme carbamoyl-phosphate synthetase 2, aspartate transcarbamoylase, and dihydroorotase (CAD), or the tetrahydrofolate cycle. It is therefore possible that in GC B cells, Asn availability tunes these nucleoside synthetic pathways, either through mTORC1 or other mechanisms. An important node in B cell nucleoside metabolism downregulated with Asn deprivation involved the enzyme which oxidises dihydroorotate, DHODH. However, the effect of Asn deprivation on mTORC1 signalling in B cells appears to vary according to setting. *In vitro* we found reduction in phosphorylation of mTORC1 target proteins. However, *ex vivo* we were surprised to find an increase following ASNase treatment in B-Asns GC B cells, accompanied by elevated phospho-Rb levels. The cause for this divergence is unclear, but may be related to differing environmental milleu, stimulation, or relative level of Asn deficiency. Whether this mTORC1 upregulation is maladaptive remains to be established.

A limitation of our study is that although we were able to perform relative quantification of Asn and Gln in LNIF, the actual concentration of these and other amino acids B cells encounter in spatially complex tissue such as the GC remains unclear.

ASNase has been a cornerstone of the treatment of leukaemia for decades, but given the results of our work, ASNS may also be a novel therapeutic target in non-malignant, autoimmune disease mediated by the GC reaction, and is deserving of future study in this context. Another aspect of ASNase is its role in promoting bacterial infection. It is interesting to speculate that its capacity to reduce environmental Asn is also effective against the protective humoral response, which is partially protected against by the expression of ASNS.

We therefore show that GC B cells activate Asn metabolism, which is required for their metabolic and functional homeostasis.

## Materials and methods

### Study design

Statistical methods were not used to pre-determine sample sizes, but sample sizes were chosen to be comparable to those reported in previous studies. Effect sizes for certain experiments were estimated through pilot studies. Data distribution was assessed using normality tests to guide the selection of appropriate statistical methods, or it was assumed to be normally distributed. Mice that lacked germinal centres in the absence of Alum spots post-immunisation were considered to have failed intraperitoneal immunisation and were excluded from analysis. Biological and technical replicates were included in all experiments, with each experiment reflecting at least two independent replicates. All experiments included in the manuscript were found to be reproducible. For in vivo experiments, sex and age were matched in experimental batches. Efforts were made to minimise potential cage and litter effects by co-housing control and experimental mice, and using littermate controls where possible, although potential cage effects could not be avoided in diet experiments. Randomisation measures applied in in vivo experiments were also carried over into in vitro and ex vivo experiments, which were conducted with cells isolated from wild-type or knockout mice. During sample acquisition, experimental and control samples were run consecutively in an alternating fashion. Other randomisation methods were not applied, as they were not relevant to the study. For some experiments, researchers were blinded, such as when mice were genotyped after the experiment. However, data collection and analysis were not blinded in most experiments because the same researchers conducted the experiments and needed genotype information to ensure the inclusion of both wild-type and knockout mice in the study.

### Mice

C57BL/6J mice were obtained from Envigo. C57BL/6N-*Asns*^*tm1c(EUCOMM)Wtsi*^/H (EM:05307) mice were obtained from the Mary Lyon Centre, MRC Harwell, UK. B6.129S6-*Eif2ak4*^*tm1*.*2Dron*^/J (*Gcn2*^-/-^)(JAX: 008240), B6.C(Cg)-*Cd79a*^*tm1(cre)Reth*^/EhobJ (JAX: 020505), B6.129P2-*Aicda*^*tm1(Cre)Mnz*^/J (JAX: 007770), and B6;129S6-*Gt(ROSA)26Sor*^*tm9(CAG-tdTomato)Hze*/J^ (JAX: 007905) mice were obtained from Jackson Laboratories. B6.SJL.CD45.1 mice were provided by the central breeding facility of the University of Oxford. Male and female mice between the ages of 6-15 weeks were used. *Asns* ^LoxP/+^ × *Cd79a*-Cre^+/-^*Asns*^*+/+*^ × *Cd79a*-Cre^+/-^*Asns* ^LoxP/+^ × *Cd79a*-Cre^-/-^and *Asns* ^LoxP/ LoxP^ × *Cd79a*-Cre^-/-^ mice were used as controls (B-WT). All phenotypes were reproduced with all control genotypes, and full genotypes are given in the raw data files.

Mice were bred and maintained under specific pathogen-free conditions at the Kennedy Institute of Rheumatology, University of Oxford. They were housed in cages that had individual ventilation and were provided with environmental enrichment. The temperature was kept between 20-24°C, with a humidity level of 45-65%. They were exposed to a 12-hour cycle of light and darkness (7 am to 7 pm), with a thirty-minute period of dawn and dusk. All procedures and experiments were performed in accordance with the UK Scientific Procedures Act (1986) under a project license authorized by the UK Home Office (PPL number: PP1971784).

### In vivo modification of asparagine levels

Experimental and control mice were intraperitoneally injected with ≈10U (500U/kg) of asparaginase from *E. coli* (Abcam, ab277068) diluted in PBS, according to experimental regime. Two main regimes were used for ASNase experiments: ‘standard’ and ‘post-GC’. In the standard schedule, ASNase was administered from one day prior to immunisation, and then every two days over a nine day period before analysis. In the post-GC schedule, only two doses of ASNase were given, at days six and eight after SRBC-immunisation or at days 6, 9 and 12 after NP-CGG immunisation, thereby targeting established GCs.

Asn-free diet or control chow (Research Diets, A05080216i and A10021Bi) was administered for 12 days before or 7 days after immunogenic challenges and maintained for the duration of the experiments. The detailed formula of diet amino acid constituents is indicated in Supplementary Table 1.

Drinking water was supplemented with L-asparagine monohydrate (Merck, cat: A7094-25G) at 1.5 g/L and administered to mice two weeks prior to immunisation and maintained for the whole duration of the experiment. Drinking water was replaced with freshly prepared Asn every 2-3 days.

### Immunisation

One ml of sterile SRBCs in Alsever’s solution (ThermoFisher, EO Labs, or TCS bioscience) were washed twice with 10ml of ice-cold PBS and reconstituted in 2-3ml of PBS, and 200μl injected intraperitoneally or intravenously. In some experiments, an enhanced SRBC immunisation method was used to maximise GC B cell yield, by immunising mice with 0.1ml SRBC on day 0 followed by a second injection of 0.2ml on day 4 or 5(49). For protein antigen immunisations, 50μg NP_(30-39)_-CGG (Biosearch Tech, cat: N-5055D-5) in PBS was mixed with Imject Alum (ThermoFisher) or Alum Hydrogel (InvivoGen, cat: vac-alu-50) at a 1:1 ratio (vol:vol) and rotated at room temperature for 30 mins (used for Imject Alum) before intraperitoneal injection in 100μl volume, or mixed vigorously with a pipette for 5 mins (used for Alum Hydrogel) before subcutaneous injection in the flanks, hock, or intraperitoneal space. 50μg NP-AECM-FICOLL (Biosearch) in PBS was mixed with PBS at a 1:1 ratio and intraperitoneally injected.

### Influenza infection

The A/HK-X31 (X31, H3N2) strain was used. Mice were anaesthetised using isoflurane and intranasally inoculated with 5×10^3^ PFU of X31 influenza A virus in PBS. Mice were closely monitored and weighed for 14 days following infection, and characteristic weight loss was confirmed in all infected mice.

### Bone marrow chimera generation

B6.SJL.CD45.1 recipient mice were administered two doses of 5.5Gy irradiation four hours apart. Mice were then intravenously injected with 4×10^6^ mixed bone marrow (BM) cells at a 1:1 ratio, isolated from age- and sex-matched CD45.2^+^ B-WT or B-Asns or *Gcn2*^-/-^ mice, and CD45.1^+^ WT donor mice. Recipient mice were maintained on antibiotics (Baytril, Bayer corporation) administered in their drinking water for two weeks. Bone marrow reconstitution was confirmed by flow cytometry of peripheral blood at 8 weeks. Asn-free diet or control chow (Research Diets, A05080216i and A10021Bi) was administered for 12 days starting from week 8, maintained for additional 9 days (total of 21 days) during immunisation with SRBC until mice were terminated at 11 weeks.

### Cell isolation

Spleens were dissociated by passing through 70μm cell strainers. For *ex vivo* GC B cell mass spectrometry, GC B cells were first enriched using the mouse Germinal Center B Cell (PNA) MicroBead Kit (Miltenyi), and then further purified by flow sorting (Live/Dead^-^ CD19^+^IgD^-^GL-7^+^CD95^+^). Total B cells from the same mouse pool were pre-enriched using CD19^+^ Microbeads (Miltenyi), then naïve B cells purified by flow sorting (Live/Dead^-^ B220^+^IgD^+^).

To isolate cells for RNA extraction, GC B cells (Live/Dead^-^CD19^+^IgD^-^CD95^+^GL-7^+^) and naïve B cells (Live/Dead^-^CD19^+^IgD^+^) were flow sorted into RLT Plus buffer (Qiagen) following pre-enrichment with the Pan B Cell Isolation Kit II (Miltenyi). B cells for culture were isolated using the Pan B Cell Isolation Kit II (Miltenyi). Purity was routinely >90% by flow cytometry.

For some experiments, untouched GC B cells were isolated using a magnetic bead-based protocol as described(50). Briefly, single cell suspensions were prepared from spleens of SRBC-immunised mice (enhanced protocol) or NP-CGG-immunised mice (scRNAseq experiment) in ice cold MACS isolation buffer (PBS with 0.5% BSA and 2mM EDTA) followed by ACK (Gibco) RBC lysis for 4 mins at 20°C. Following washing, cells were labelled with anti-CD43 microbeads (Miltenyi) and biotinylated antibodies against CD38 and CD11c (both eBioscience, clones 90 and N418 respectively) and IgD (Thermofisher, clone 11-26c.2a). Then, cells were incubated with anti-biotin Microbeads (Miltenyi), and subsequently run through an LS column (Miltenyi). GC B cell purity was typically around 90%. For scRNAseq, GC B cells were further purified using fluorescence-activated cell sorting (FACS).

### Cell culture

Total B cells were isolated as described above. B cells were cultured at 1-3×10^6^ cells/ml in RPMI1640 (custom product from Cell Culture Technologies, Gravesano, Switzerland, lacking glutamate, aspartate, glutamine, and asparagine), supplemented with 1mM pyruvate, 10mM HEPES, 100 IU/ml penicillin/streptomycin and 50μM 2-mercaptoethanol, and 10% dialysed FBS (Gibco), Aspartate (Asp) (150μM), Glutamate (Glu) (140μM), GlutaMAX (ThermoFisher or Sigma) (2mM), indicated concentration of Asn, except where mentioned otherwise. B cells were stimulated with agonistic anti-CD40 (5μg/ml, FGK45.4, functional grade, Miltenyi), recombinant IL-4 (1,10 or 50ng/ml, Peprotech), LPS (10μg/ml, Merck) or CpG (100nM, ODN1826, Miltenyi).

For proliferation experiments, cells were labelled with CellTrace Violet (ThermoFisher) according to manufacturer’s instructions before stimulation. For stable isotope labelling, ^15^N(amide)-asparagine (485896, Merck) (400μM), ^15^N(amide)-glutamine (NLM-557, Cambridge Isotope Laboratories) (2mM), ^15^N(amino)-glutamine (NLM-1016, Cambridge Isotope Laboratories) (2mM), or ^13^C_5_-glutamine (CLM-1822, Cambridge Isotope Laboratories) (2mM) were used, replacing their unlabelled molecule in RPMI1640.

For nucleoside rescue experiment, adenosine (Fluorochem cat: F093333), thymidine (Fluorochem cat: F078885), uridine (Fluorochem cat: F078878), cytidine (Fluorochem cat: F226675) and guanosine (Merck, cat: G6264-5G) were prepared freshly in water and added to culture media at a final concentration of 30μM or 100μM.

Halofuginone hydrobromide (Bio-techne, cat no: 1993) was reconstituted in DMSO and used at 2nM and 5nM in the culture. Dimethyl 2-oxoglutarate (DM-OG) was purchased from Merck (cat no: 349631-5G) and added to the culture medium at 2mM and 4mM final concentrations.

### Ex vivo lymph node slice culture

Inguinal lymph nodes (LNs) were harvested from B-WT or B-Asns mice immunised with NP-CGG (precipitated in Alum hydrogel) subcutaneously on both flanks, at day 14. They were then removed from surrounding fat by dissection and mild detergent wash (<1 sec) in 0.01% digitonin solution (Thermo Scientific) and embedded in 6% w/v low melting point agarose (Lonza) in 1x PBS. 300μm thick slices were cut using the Precisionary Compresstome® VF-210-0Z following manufacturer’s instructions with a blade speed of 4 and oscillation of 6. The buffer tank was filled with ice cold PBS containing 1% Pen/Strep. Slices were removed from surrounding agarose and placed in a 24 well plate in 500μl of Asn-replete or Asn-free complete RPMI media in duplicate. LN slices were allowed to rest and equilibrate in the media for 1 hour at 37˚C and 5% CO2 before transferral into fresh media and incubation for 20hrs. Following culture, slices were collected, duplicate slices from same lymph nodes pooled, and slices disrupted into PBS containing 2% FBS and 2mM EDTA using the tip of an insulin syringe plunger to generate a cell suspension in a 96-well U bottom plate. Cells were then stained with viability dye and surface antigens. Following washes, cells were fixed in fresh 4% PFA (Cell Signalling) for 15 mins at 20°C and permeabilised in methanol for intracellular staining and subsequently run on an Aurora flow cytometer (Cytek).

### Ex vivo single cell competitive Asn uptake assay

We followed the recently published QUAS-R protocol with certain modifications(27). Briefly, spleens were harvested 8 days post SRBC immunisation and dissociated through 70μm cell strainers. Red blood cells were lysed with ACK lysis buffer and single cell suspensions were incubated with Zombie NIR (BioLegend) and Fc block in PBS on ice for 30 minutes followed by surface staining in FACS buffer for 30 minutes on ice. Hanks’ Balanced Salt Solution HBSS (ThermoFisher) supplemented with Click-iT homopropargylglycine HPG (ThermoFisher) at 400μM and L-asparagine monohydrate (Merck) at increasing concentrations (0, 10, 40, 100, 400, 4000μM) were warmed at 37°C. Cells were resuspended in HBSS and 50μl of suspension was plated and incubated at 37°C for 10 minutes. Control cells incubated at 4°C were kept on ice for background detection. HPG-containing media supplemented with each Asn dilution was added and cells were further incubated for 3 minutes, before immediate fixation with PFA at 4% for 25 minutes at room temperature. Cells were washed and permeabilised with 1× saponin-based permeabilization buffer (ThermoFisher) for 20 minutes at room temperature before adding the click chemistry reaction mix, using the Click-iT™ Plus OPP Alexa Fluor™ 647 Protein Synthesis kit due to the shared Click Chemistry of HPG with OPP and incubating for 30 minutes at room temperature. Cells were washed and resuspended in FACS buffer for flow cytometry acquisition.

## Supplementary Material

Supplementary Materials

## Data Availability

For the purpose of open access, the author has applied a CC BY-ND public copyright license to any Author Accepted Manuscript version arising from this submission. The scRNA sequencing data generated in this study has been deposited to GEO under accession code GSE277338. All data needed to evaluate the conclusions in the paper are present in the paper or the Supplementary Materials. Tabulated underlying data for all figures can be found in supplementary data file S2.

## One sentence summary

Asparagine metabolism is a critical regulator of germinal centre B cell function.
