# Supplementary material for: Asparagine availability controls germinal centre B cell homeostasis
Source: Sci Immunol. Author manuscript; Available in PMC 2025 Mar 12. (PMC7617476; doi:10.1126/sciimmunol.adl4613)
Supplement: Supplementary Materials [file EMS203580-supplement-Supplementary_Materials.pdf]

Supplementary materials

Supplementary materials and methods
Supplementary figures S1-S11

Supplementary data file S1. Metabolomics data
Supplementary data file S2. Raw data excel file

!K* E#;)3,!1;0#;7,K!F+0!E>!:!*7--1!_>INd/D,I 23>IS^ 9EHT]/a!#36!3#e$7D,I/!:!*7--1!
_>INd/D,I/a(
3K* @7J07173;#;)$7!F-+8!*K;+G7;0K!?)1;+,0#G!#36![%#3;)F)*#;)+3!+F!>Id^!,7+G7;0)*!G7#3!
F-%+071*73*7!)3;731);K!_,2'Da!)3!D/,I!3#e$7!#36!E>!:!*7--1!_3•X!G)*7a.!#1!)3! (!I#;#!
07J07173;#;)$7!+F!;8+!)367J73673;!7MJ70)G73;1(!!
<K* @7J07173;#;)$7!)GG%3+F-%+071*73*7!)G#,71!+F!1J-73)*!E9+1G!W:LT)3g7*;76!5)*6#T
;6U+G#;+!G)*7.!37,#;)$7!*+3;0+-!F+0!&*J ! U3 (!@7J07173;#;)$7!+F!;8+!)367J73673;!
7MJ70)G73;1(!L*#-7!A#0u!Nff G(!
DK* Z%#3;)F)*#;)+3!+F!WW!)9A'&K>J< !#36!I'&K*J3 (!/#*?!6#;#!J+)3;!07J07173;1!#3!
)36)$)6%#-!E>(!@7J07173;#;)$7!+F!;8+!)367J73673;!7MJ70)G73;1(!
FK* E#;)3,!1;0#;7,K!+F!-),?;!&+37!_>I^\ 01>b>@V23a!#36!6#0<!&+37!_>I^\23>b>@V01a!E>!:!
*7--1(!
IK* DGG%3+A-+;!+F!5LRL!)3!G#,37;)*!A7#6!1+0;76!E>!:!*7--1!#36!3#e$7!*7--1.!F0+G!8)-6!
;KJ7!G)*7!)GG%3)176!8);?!L@:>!#;!6#K!f!#36!6#K!V.!8);?!#3#-K1)1!#;!6#K!NQ(!
@7J07173;#;)$7!+F!;8+!)367J73673;!7MJ70)G73;1(!:7#*#;)3!)1!%176!#1!#!-+#6)3,!
*+3;0+-(
;K* >7--IDo/!)GG%3+F-%+071*73*7!)G#,71!+F!#!E>!F0+G!#!;+31)-!?),?-),?;)3,!5LRL!)3!
>IQN/!HB.!>IQN9!IB.!D,I /!3#e$7.!#36!2B:N/!J-#1G#!*7--!3)*?71(!
LK* Z%#3;)F)*#;)+3!+F!G7#3!F-%+071*73*7!)3;731);K!_2'Da!+F!5LRL!)3!#07#1!1?+D8[3!)3!
)3*-%6)3,!>IS/!U!*7--1(!/#*?!6+;!07J07173;1!#0#36+G-K!17-7*;76!E>!8);?!#!
07J07173;#;)$7!IB.!HB!#36!1%00+%36)3,!D,I/3#e$7!:!*7--!F+--)*-7!_3•QN!E>1!)3!;+;#-.!
J++-76!F0+G!;?077!;+31)-1a(!@7J07173;#;)$7!+F!;8+!)367J73673;!7MJ70)G73;1(!
8K* >7--IDo/!)GG%3+F-%+071*73*7!)G#,71!+F!#!E>!F0+G!#!;+31)-!?),?-),?;)3,!LH>N5X!
_5L>UQa!7MJ0711)+3!)3!>IQN/!HB.!>IQN9!IB.!#36!1%00+%36)3,!D,I/!3#e$7!:!*7--!
F+--)*-7(!L*#-7!A#0!NfflG(
!
L;#;)1;)*#-!1),3)F)*#3*7!8#1!67;70G)376!AK!%3J#)076!;B#)-76!;!;71;!_:a!;8+ T8#K!5RPo5!
8);?!L)6#<n1!G%-;)J-7!;71;)3,!*+007*;)+3!_!#36!+37T8#K!5RPo5!8);?!U%<7Kn1!G%-;)J-7!
;71;)3,!*+007*;)+3!4 a(!I#;#!#07!J07173;76!#1!;?7!G7#3!^j T!L/2(!
!
!
!
!

!
